# Pathophysiological, Neuropsychological, and Psychosocial Influences on Neurological and Neuropsychiatric Symptoms of Post-Acute COVID-19 Syndrome: Impacts on Recovery and Symptom Persistence

**DOI:** 10.3390/biomedicines12122831

**Published:** 2024-12-13

**Authors:** Alex Malioukis, R Sterling Snead, Julia Marczika, Radha Ambalavanan

**Affiliations:** Self Research Institute, Broken Arrow, OK 18452, USA

**Keywords:** post-acute COVID syndrome, long COVID, pathophysiology, neurological symptoms, cognitive dysfunction, psychological, neuropsychological, psychosocial

## Abstract

Although the impact of post-acute COVID-19 syndrome (PACS) on patients and public health is undeniably significant, its etiology remains largely unclear. Much research has been conducted on the pathophysiology, shedding light on various aspects; however, due to the multitude of symptoms and clinical conditions that directly or indirectly define PACS, it is challenging to establish definitive causations. In this exploration, through systematically reviewing the latest pathophysiological findings related to the neurological symptoms of the syndrome, we aim to examine how psychosocial and neuropsychological symptoms may overlap with neurological ones, and how they may not only serve as risk factors but also contribute to the persistence of some primary symptoms of the disorder. Findings from our synthesis suggest that psychological and psychosocial factors, such as anxiety, depression, and loneliness, may interact with neurological symptoms in a self-reinforcing feedback loop. This cycle seems to be affecting both physical and psychological distress, potentially increasing the persistence and severity of PACS symptoms. By pointing out this interaction, in this review study, we attempt to offer a new perspective on the interconnected nature of psychological, psychosocial, and neurological factors, emphasizing the importance of integrated treatment approaches to disrupt this cycle and improve outcomes when possible.

## 1. Introduction

PACS refers to a spectrum of symptoms and conditions that persist following the acute phase of infection caused by the severe acute respiratory syndrome coronavirus-2 (SARS-CoV-2) [1]. The terminology surrounding this condition is extensive, with various names such as long-haul COVID-19, post-COVID-19 conditions (PCCs), or post-acute sequelae of SARS-CoV-2 infection (PASC) often used interchangeably. In this review, we adopt the term post-acute COVID-19 syndrome (PACS) to describe the symptomatology under investigation. As noted by Ezpeleta et al., this term is preferable as it carries fewer negative implications compared to the alternatives that suggest SARS-CoV-2 may linger in the body for extended periods, a claim unsupported by the current evidence [2].

It is estimated that PACS has impacted nearly 400 million individuals since the start of the pandemic [3]. Although still the subject of extensive global research, this condition is recognized as an intricate, multisystemic disorder that can influence almost every organ system, including the nervous, cardiovascular, gastrointestinal, and endocrine systems [3,4,5]. While certain individuals, such as older adults, females, those with lower socioeconomic status, and individuals with pre-existing health conditions or mental health disorders, are most at risk for developing long-lasting symptoms after a COVID-19 infection, the syndrome can affect people across all age groups, including children and older adults, and across diverse races, ethnicities, genders, and health backgrounds. Importantly, PACS has been documented following both severe and mild cases of acute COVID-19 [6,7]. The commonly reported symptoms include fatigue, shortness of breath, and brain fog, but they can also extend to joint pain, dizziness, chest discomfort, sleep disturbances, and altered senses of smell or taste. Additional symptoms may involve headaches, gastrointestinal issues, and psychological impacts such as anxiety and depression [8]. The symptoms persist for more than 12 weeks following the onset of COVID-19 and cannot be explained by any alternative diagnosis. The condition is identified through clinical evaluation, as there is currently no definitive biomarker to confirm its presence [9,10].

In this study, we will primarily focus on the neurological, neuropsychological, and neuropsychiatric symptoms of PACS. Our review presents a new angle on PACS by exploring the complex interactions between psychological, psychosocial, and neurobiological factors, which, to the best of our knowledge, have not been thoroughly explored in previous studies. By examining how these interconnected symptoms may create a feedback loop, where the physical and psychological conditions exacerbate one another, we aim to offer a more holistic perspective on the disorder. This integrated approach, which includes psychosocial and psychological dimensions, offers new insights into the persistence and complexity of PACS, going beyond typical studies focused mainly on the pathophysiological aspects.

## 2. Methods

### 2.1. Search Strategy

An online search of the existing published literature was conducted to identify studies examining the multifactorial causes of PACS, covering the period from 2020 to 2024. This study was registered in the OSF; registration DOI https://doi.org/10.17605/OSF.IO/RCGH2. We used the PRISMA guidelines as a general framework to guide our review [11]. In doing so, five scientific literature databases—namely, Google Scholar, Scopus, PubMed, PsycINFO, and the ACM Digital Library—were electronically searched. To ensure a comprehensive retrieval of related articles, we employed a strong set of keywords covering essential and supporting terms related to PACS, its physiological and psychological causes, and treatment strategies (see Table 1). The search for relevant articles was conducted between 5 September 2024 and 15 November 2024, and was limited to English-language publications.

Thematic and keyword search results were then manually screened for relevance to PACS and its underlying mechanisms. Each study was carefully examined, with particular attention to recent findings on the neuropathology of PACS, as well as the neuropsychological and psychosocial factors affecting the disorder.

### 2.2. Eligibility Criteria

The inclusion criteria for this review were peer-reviewed journal articles, conference papers, and review articles presenting empirical findings, theoretical frameworks, or innovative methodologies related to PACS or post-COVID-19 conditions, published in English between 2020 and 2024. Studies focused specifically on PACS or post-COVID-19 symptoms were included. The exclusion criteria included studies that focused on populations without a PACS diagnosis, such as healthy controls or individuals with unrelated comorbidities. Studies that did not address PACS-related neurological, neuropsychological, or psychosocial outcomes were excluded. Additionally, studies focused on disorders unrelated to PACS, such as general neurological diseases not linked to COVID-19, were excluded.

### 2.3. Data Synthesis

**Figure 1:**
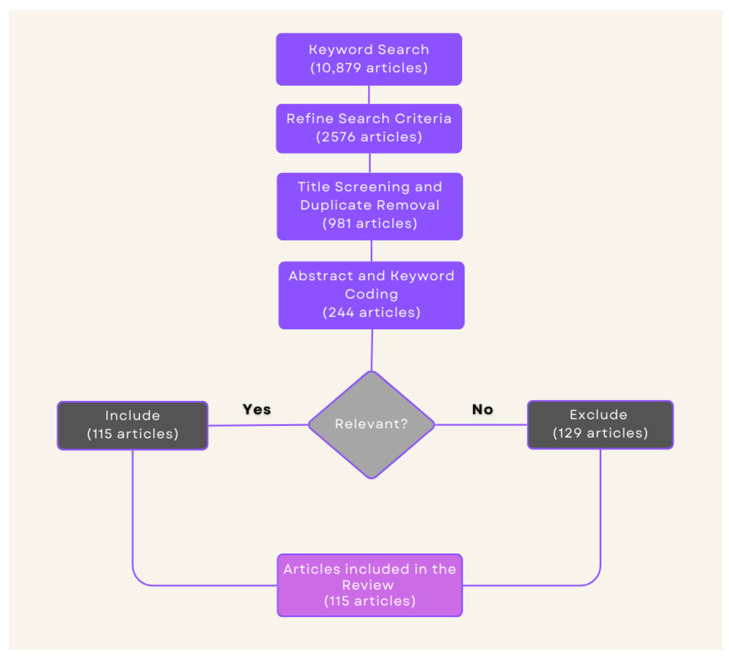
Flow diagram of literature search and selection process.

The authors performed an independent screening of this article based on the inclusion and exclusion criteria described previously. Initially, a total of 10,879 articles were retrieved from keyword searches conducted across the databases mentioned above. By limiting our selection to peer-reviewed journal articles published in English, for which full texts were available, and by removing duplicates, the total was reduced to 2576 articles. In conducting this review, the first batch of retrieved publications served as the basis for screening to choose those that fulfilled our set criteria. In the first phase, all articles underwent an initial screening based on their titles to ensure consistency and relevance to our research goals. Following this initial screening, 981 were selected for further evaluation. The second step in preliminary coding involved the reviewing of abstracts, keywords, and, when necessary, introductory sections of the articles with an aim to determining which had close relevance to our current research focus. This narrowed down our selection to a subset of 244 articles. Lastly, in the final stage of screening, the articles that were remaining were thoroughly read through with an objective of determining their applicability and relevance within the scope of our study. Accordingly, a final set of 115 articles that substantially contributed to our research objectives and met the set inclusion criteria was identified and retained. The process for article retrieval, screening, and selection is shown in Figure 1.

## 3. Neurological, Neurocognitive, and Neuropsychiatric Symptomatology of PACS

While COVID-19 is primarily recognized as a respiratory illness, it is also closely linked to a variety of neurological and neuropsychiatric manifestations due to its impact on both the central nervous system (CNS) and peripheral nervous system (PNS). In the context of PACS, the extensive range of symptoms, including numerous neurological and neuropsychological issues, has led clinicians worldwide to suggest that it resembles a neurological disorder in many respects [12].

### 3.1. Neurological Symptoms

PACS manifests a wide range of neurological and neuropsychological symptoms, highlighting the intricate relationship between the CNS and the PNS after a SARS-CoV-2 infection 9 (see Table 2). Notably, many of these neurological symptoms often overlap with issues impacting various organ systems. Furthermore, some nonspecific symptoms that we will examine may indicate secondary effects arising from underlying conditions in the endocrine, autoimmune, or psychiatric and psychological areas.

One of the most severe and persistent symptoms reported in the literature is fatigue. This condition affects a considerable proportion of PACS patients, with evidence indicating that up to 58% experience long-lasting fatigue, which can negatively influence daily functioning and overall well-being [13,14]. Sleep disturbances, particularly insomnia, are also prevalent among PACS patients and are closely associated with cognitive impairments and emotional distress. Insomnia affects up to 30% of this population, with these disruptions in sleep quality potentially exacerbating fatigue and affecting cognitive performance [15,16].

Headaches are another commonly documented symptom, with varying severity and frequency. Observational studies report that up to 44% of patients continue to experience headaches months post-infection. These headaches are often resistant to standard treatment and are associated with other symptoms, such as concentration problems and heightened sensory sensitivities [17,18].

In addition, symptoms of dizziness and vertigo are also frequently observed, affecting up to 5% of PACS patients, and can present as episodes of lightheadedness and balance impairment, which can have a negative impact on mobility and quality of life. These symptoms may be indicative of autonomic dysfunction, which is commonly reported in PACS. More specifically, it has been observed that post-COVID-19, up to 14% of patients present with postural orthostatic tachycardia syndrome (POTS), a manifestation of autonomic dysfunction, which is characterized by symptoms such as increased heart rate upon standing, palpitations, chest pain, lightheadedness, and fainting episodes, illustrating the widespread autonomic impact of SARS-CoV-2 infection. As with many other symptoms of the disorder, it is still unclear whether POTS is a direct symptom of PACS or if COVID-19 infection acts as a trigger in individuals who may already be predisposed to developing dysautonomia. In these cases, COVID-19 might not directly cause dysautonomia but instead exacerbates or activates an underlying susceptibility [19,20].

Another syndrome that overlaps with PACS, and shares similarities with POTS, is myalgic encephalitis/chronic fatigue syndrome (ME/CFS). Common ME/CFS-like symptoms, experienced by up to 51% of patients, include severe muscle weakness, joint pain, dizziness, and cognitive dysfunction, often occurring alongside unrefreshing sleep. These findings suggest a potential shared pathophysiology between PACS and ME/CFS, contributing to the complexity of symptom management and quality of life deterioration [21].

Finally, other common symptoms, while not entirely neurological and also frequently observed in neuropsychiatric and psychological disorders, are gastrointestinal (GI) disturbances. We chose to explore these symptoms further because they have become a notable area of focus, partly due to recent studies suggesting the involvement of the gut–brain axis and the bidirectional relationship between the CNS and the gut [22,23,24]. GI symptoms are reported in a significant portion of PACS patients, with a systematic review and meta-analysis revealing that 22% experience GI issues—nearly double the 12% observed during the acute infection phase. Common symptoms include abdominal pain, nausea, vomiting, loss of appetite, loss of taste, diarrhea, dyspepsia, and irritable bowel syndrome (IBS). These GI symptoms often occur alongside systemic and neuropsychiatric issues; for instance, gut dysbiosis in PACS patients has been associated with fatigue, cognitive impairments, and neuropsychiatric symptoms such as anxiety and depression [25,26,27,28].

### 3.2. Neurocognitive and Neuropsychological Symptoms

Building on the neurological symptoms discussed in the previous section, neurocognitive symptoms in PACS reflect the complex interplay between the neurological, neuropsychological, and psychological factors. The emerging literature over the past four years has begun to shed light on how these symptoms affect cognitive processes, including attention, memory, and executive functioning (see Table 3). In this chapter, we will review the current research to better understand these cognitive challenges and their impact on daily life.

Regarding the prevalence of the symptoms, a systematic review by Tavares-Júnior et al. found that cognitive impairment was observed in 21% to 65% of COVID-19 survivors who had been hospitalized, with assessments conducted 12 weeks or more after the initial infection [29].

Following COVID-19 infection, numerous studies have documented a range of cognitive challenges reported by patients months post-recovery. Common complaints, from up to 47% of patients, include persistent fatigue as well as brain fog, and difficulties with attention and memory [30,31,32]. The results from neuropsychological testing indicate that individuals with a recent SARS-CoV-2 infection often exhibit impaired global cognitive function, leading to notable challenges in concentration and sustained attention, among other difficulties [33]. The research has consistently shown that memory problems, including deficits in working memory, verbal memory, and visual memory, are also prevalent among those recovering from COVID-19, affecting up to 40% of PACS patients [34,35,36].

Additionally, studies indicate that up to 59% of individuals with PACS demonstrate executive function deficits and slowed information processing speed, factors which can also complicate everyday functioning and well-being [37,38]. Language difficulties are also frequently reported among adults with PACS, with studies showing poorer performance in areas such as discourse informativeness, verbal recall, and fluency. Cummings, for instance, found that while adults with PACS can produce grammatically correct speech, their discourse often lacks informativeness, a problem linked to executive dysfunctions like impaired planning. Memory deficits further contribute to reduced informativeness, as seen in significant recall challenges among participants. Additionally, the same study indicates that word-finding difficulty is a common complaint, affecting 93% of surveyed adults, despite normal naming task performance. Verbal fluency tasks reveal further deficits, especially in letter and category fluency, which when coupled with the other language difficulties, highlight the cognitive–communication disruptions experienced by individuals with PACS [39].

Brain fog, a term frequently discussed in studies on PACS, encompasses a combination of the aforementioned symptoms along with other psychological phenomena [40]. More specifically, in post-COVID-19 syndromes, brain fog is recognized as a symptom cluster characterized by fatigue, headaches, word-finding difficulties, memory impairment, inattention, short-term memory loss, and reduced mental acuity. This cluster significantly affects cognition, concentration, and sleep and is associated with adverse psychological and psychomotor outcomes [41,42].

These findings, among others, indicate that cognitive challenges such as attention, memory, and executive dysfunction are not merely isolated symptoms but instead reflect the broader neurological substructures affected, either directly or indirectly, by the virus.

### 3.3. Neuropsychiatric Symptoms

While examining the literature on psychiatric symptoms after COVID-19 infection, we cannot overlook the overlapping nature of these symptoms with other domains and the role they play in the complex interactions of PACS. Once again, the research suggests that the psychiatric symptoms in PACS are not isolated occurrences but are deeply interwoven with both neurological and psychosocial variables, influencing the broader spectrum of symptoms experienced (see Table 4).

Anxiety and depression emerge as some of the most frequently reported psychiatric symptoms, with studies highlighting that these issues can persist for extended periods post-infection, affecting up to 29.6% and 35% of individuals, respectively. The symptoms often linger for weeks to months and show higher prevalence among females and those with a history of psychiatric disorders, suggesting that COVID-19 may act as a trigger or an exacerbating factor for pre-existing mental health vulnerabilities. This persistent presence of anxiety and depression contributes to the complexity of PACS, as these symptoms interact with other cognitive and physical challenges, potentially increasing the overall burden on patients [43,44].

PTSD, too, is a prominent psychiatric concern in the context of PACS, with multiple studies noting substantial rates of PTSD symptoms among COVID-19 survivors. Although prevalence estimates vary, with up to 16% of patients experiencing symptoms of the disorder, the high occurrence of PTSD could reflect the significant psychological distress that individuals may experience following severe or life-threatening COVID-19 infections. Factors such as hospitalization, prolonged isolation, and the psychological toll of the pandemic itself may also contribute to the persistence of these symptoms [45,46,47,48].

Sleep disturbances—observed both in the context of neurological symptoms and from a psychiatric perspective—also constitute a common complaint among patients. Mekhael et al. utilized wearable health devices to examine the impact of PACS on sleep quality and found that individuals with a history of COVID-19 infection exhibited significant alterations in sleep architecture. Specifically, these patients experienced reduced total sleep duration and less deep sleep compared to matched controls, irrespective of demographic factors and symptom severity [49]. Insomnia and hypersomnia frequently appear as prevalent symptoms, with disrupted sleep potentially acting as both a direct response to the virus and as a secondary effect of co-occurring psychiatric conditions, such as anxiety or depression. Notably, sleep problems are often among the most pervasive symptoms of PACS, impacting mental health, cognitive function, and overall well-being [50,51,52]. Interestingly, in their recent paper, Schilling et al. concluded that disturbed sleep patterns could even potentially predict an increased risk of developing post-acute sequelae of COVID-19 [53].

The recent research highlights another dimension of psychiatric disorders related to COVID-19, specifically psychotic symptomatology. These may include delusions, hallucinations, disorganized thoughts, and confusion and can affect up to 4% of the patients. Cases vary in duration, lasting from a few days to several weeks, with some extending up to 90 days [54,55]. Studies by Ferrando et al. and Smith et al. have documented symptoms such as paranoia, megalomania, mystical beliefs, and catatonia, affecting predominantly male patients with a mean onset age of 43.9 years. Long-term symptoms were also noted in some patients months after initial infection [54,56,57].

In conclusion, the psychiatric symptoms of the syndrome are complex and interconnected with other neurological and psychological factors. Of these, anxiety, depression, and PTSD are the most frequent and often aggravate the cognitive and physical difficulties, creating a vicious circle that further escalates the severity of both mental and physical suffering. Moreover, common sleep disturbances also interact with these psychiatric symptoms, increasing the overall burden of PACS. These overlapping domains seem to contribute to the multifaceted nature of PACS, where certain psychiatric manifestations are not merely symptoms themselves but also potential risk factors for the development or exacerbation of other symptoms. In the following sections, we will explore the neurobiology and psychological aspects of these symptoms and how they may contribute to the broader challenges of PACS.

## 4. Pathophysiology

The pathophysiological and neurophysiological mechanisms underlying the resulting neurological, neuropsychological, and neuropsychiatric symptomatology are under active investigation. The extant literature puts forward a variety of neurobiological mechanisms that could be implicated in these symptoms, and it may well be that an interplay among these processes could drive the diverse manifestations seen thus far.

### 4.1. Immune–Inflammatory Signaling and Cytokine Response

According to the low-grade inflammation theory, the pathogenesis of PACS is promoted by a persistent, low-level inflammatory responses rather than the intense hyperinflammation usually seen in acute COVID-19 cases. This chronic, mild immune activation subtly seems to perturb cellular and systemic functions, contributing to the diversity of symptoms in PACS, such as fatigue, cognitive dysfunction, and mood disturbances. In contrast to the acute and life-threatening inflammation presented during the initial phases of infection, persistent low-grade inflammation is similar to para-inflammatory mechanisms observed in chronic viral infections and post-infectious syndromes. Manifestation of this dysregulation of immune response may lead to the sustained activation of microglia and release of cytokines, thereby providing grounds for the development of neuropsychiatric symptoms, especially through changes in susceptible brain areas, such as the limbic system [58,59].

Cytokines such as TNF-α (tumor necrosis factor), IL-6 (interleukin-6), and IL-1β are implicated not only in the inflammatory response to COVID-19 but also in the development of conditions like major depressive disorder and Alzheimer’s disease. Such cytokines may contribute to dysregulated stress responses, mood changes, and cognitive dysfunction in post-infection individuals. The fact that elevated levels of these cytokines are often seen in COVID-19 patients suggests that inflammation could play a significant role in the cognitive and emotional disturbances observed in PACS [51].

Further supporting this hypothesis, one narrative review article underlines some neuropsychiatric manifestations related to immune–inflammatory signaling in COVID-19, suggesting that systemic inflammation per se can cause psychiatric presentations even in the absence of direct viral CNS invasion [48]. Indeed, significant correlations exist between levels of TNF-α, IL-6, IL-1β, IL-10, IFN-γ, and CCL2 with cognitive and behavioral changes in COVID-19 patients, thus showing generalized neuroinflammation. This dysregulation, especially the pro-inflammatory cytokines, can affect the integrity of the blood–brain barrier by allowing immune cells to penetrate the central nervous system, which impairs neurotransmission. Accordingly, such immune-mediated alterations may subsequently give rise to neuropsychiatric manifestations of depression, anxiety, and cognitive dysfunction [60,61]. The immune response against COVID-19 is thought to result in a high-degree inflammatory response by the release of both Th1 and Th2 cytokines. This excessive response could lead to an exaggerated state of inflammation in the body, hence facilitating different kinds of organ injuries. Within the central nervous system, this can lead to immune-mediated disorders characterized by delirium, cognitive dysfunction, and changes in mood [62]. Indeed, elevated levels of pro-inflammatory biomarkers, like IL-6 and CCL2, have been found to relate to changes in cerebral activity, particularly in those regions—such as the sub-genual cingulate cortex—critical for maintaining mood [63].

### 4.2. Prolonged Neuroinflammation and Psychiatric and Neurocognitive Symptoms

Some studies have suggested that rather than being a mere residue of the acute infection, many psychiatric symptoms in PACS may appear and increase post-infection as a consequence of neuroinflammation persisting for long periods [64,65]. Such an effect might depend importantly on the activation of microglia and reactive astrogliosis. The mentioned glial cell responses can disrupt glutamate signaling and elevate the risk of both new-onset psychiatric disorders and exacerbation of pre-existing conditions. This ongoing neuroinflammatory response possibly indicates a complicated way in which COVID-19 maintains its effects on brain function even long after infection. Speculations are that this greatly contributes to the cognitive and emotional symptoms seen in patients with PACS [66].

### 4.3. Viral Pathways to the CNS and Brainstem Dysfunction

COVID-19’s impact on the CNS may also be linked to direct or indirect viral entry. While the olfactory bulb has been identified as a potential entry point for the virus, autopsy findings have shown the presence of viral RNA in the brainstem, a region with high ACE2 receptor expression. This suggests that SARS-CoV-2 may infect the brainstem and, thus, may be involved in neurodegenerative processes. Not all cases, however, show detectable viral RNA in the brainstem, for which reason, researchers also believe that neurological damage is largely immune-mediated rather than via the direct infection of brain tissue. This notion receives further support from the fact that symptoms related to PACS, including tiredness and cognitive confusion, are in line with symptoms of chronic fatigue syndrome, in which abnormalities in the brain stem are often observed [67,68].

### 4.4. Neuroimaging Findings

**Figure 2:**
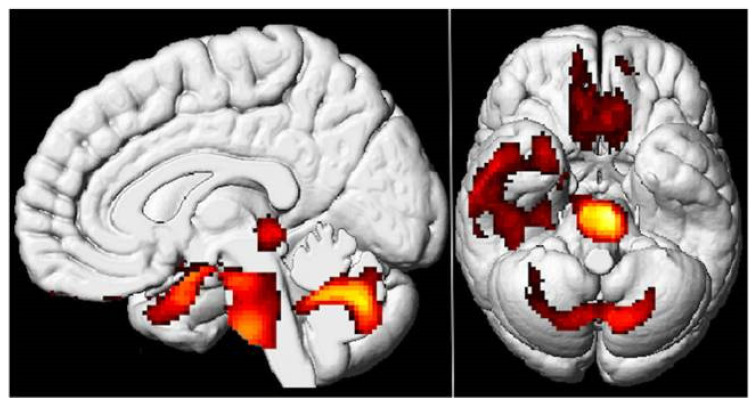
The 18F-FDG PET demonstrating hypometabolism in the bilateral rectal/orbital gyrus, the right temporal lobe, the bilateral pons/medulla brainstem, and the bilateral cerebellum in patients with PACS. Reproduced with permission from reference Guedj et al. [72].

Several recent neuroimaging studies have indeed underlined the long-term neurological burden of COVID-19 and showed both structural and functional alterations in the brain. Indeed, several studies using different imaging modalities, such as FDG-PET and MRI (see Figure 2), have suggested that COVID-19 may induce frontoparietal hypometabolism and a reduced gray matter volume of the orbitofrontal cortex and the parahippocampal gyrus. These findings resonate with the cognitive and emotional disturbances noted in patients suffering from PACS, which frequently manifest as challenges in attention, memory, and the regulation of mood [69,70,71].

Researchers have therefore speculated that dysfunction here might be contributing to the chronic fatigue and brain fog commonly reported by patients with PACS, which somewhat echoes related studies into chronic fatigue syndrome where the brainstem has also been implicated. This association of cognitive symptoms with brainstem dysfunction is further supported by the diffusion-weighted (DWI) studies which have shown microstructural changes in the thalamus, another region implicated in CFS [72,73].

Various neuroimaging studies have also invariably pointed to changes not only in the brainstem and thalamus but also in different white matter tracts connecting far-reaching cortical regions. These include the corona radiata, corticospinal tract, corpus callosum, and arcuate fasciculus, among others. These white matter changes imply an interruption to communicating streams across different brain regions; these, in turn, can be considered responsible for the cognitive–affective symptoms constituting PACS [70].

Supporting these structural findings, the recent narrative review by Theofilis et al. summarized important neurophysiological findings with significant changes in the functional and structural features of the brains of subjects affected by PACS [74]. In a longitudinal EEG study, patients recovering from COVID-19 presented increases in regional current density and connectivity in the delta band, associated with executive function impairment two months post hospitalization [75]. Further neuroimaging studies have also been able to show frontoparietal hypometabolism, possible dysfunction in the locus coeruleus, gray matter reduction in the orbitofrontal cortex and parahippocampal gyrus, alongside global brain volume loss, following COVID-19 infection. This would suggest that COVID-19 infection may result in long-lasting structural changes to those parts of the brain involved in cognitive and emotional processing [69,76].

### 4.5. Gut-Brain Axis

The emerging research has suggested that the gut–brain axis may also play a role in the neuropsychopathology of PACS. SARS-CoV-2 can disrupt the gut microbiome, leading to neuroimmune interactions that potentially contribute to prolonged cognitive and mood disturbances. The virus uses ACE2 receptors, found abundantly in both the gut lining and the brain’s blood vessels, to gain entry into these regions. This interaction could facilitate the disruption of the blood–brain barrier, allowing inflammatory and neurotoxic molecules to cross into the brain and facilitate symptoms such as memory deficits and mood swings. The gut–brain axis research has determined that the permeability of the gut is increased due to stress and illness, a condition popularly called “leaky gut”, through which toxins may enter the bloodstream. These products, once in the brain, can further increase cognitive symptoms like brain fog and amnesia, and even influence mood. Moreover, gut dysbiosis, or an imbalance in the gut microbiota, may result in chronic inflammation and immune activation, further promoting neuropsychiatric symptoms in PACS. The gut–brain axis disruption model contrasts with the hypothesis of direct viral entry into the brain and thus offers an indirect route for neurocognitive symptoms associated with PACS [77,78].

### 4.6. Impact of Viral Variants and Vaccination on PACS

To further explore the variability in post-COVID-19 presentations, it could also be helpful to consider the influence that different viral variants may have on the severity and persistence of symptoms in PACS patients.

Investigations have indicated that variants of SARS-CoV-2 may also affect the presentation and likelihood of PACS. An analysis contrasting individuals infected with the wild-type virus (March–December 2020) with those infected by the Alpha variant (January–April 2021) uncovered a change in symptomatology, especially within the neurological and cognitive/emotional areas. Despite this diversity, persistent fatigue and breathlessness continued to be the symptoms most often reported. These data therefore support the concept that a switch in tropism and/or other viral properties could partially explain the phenotypic differences among post-COVID-19 symptoms [79].

In a similar manner, another study analyzed the relationship between SARS-CoV-2 variant and PACS risk in 7699 hospitalized patients from six centers. Researchers found that patients infected with the wild-type virus exhibited the highest prevalence of symptoms, with fatigue, brain fog, and respiratory symptoms as the most common manifestations. Interestingly, Omicron had a lower risk compared to the variants from the earlier period, Alpha and Delta. The mediation analyses showed that part of the decrease in the risk was mediated through the weaker severity of acute disease with lower rates of ICU admission [80]. Further research has identified symptom clusters with distinct variants. For example, an unsupervised analysis of post-COVID-19 profiles revealed three primary symptom clusters—cardiorespiratory, central neurological, and severe multi-organ dysfunction. The frequency and severity of these clusters were variant-dependent. For instance, the Delta variant elicited severe symptoms of multi-organ dysfunction, whereas neurological symptoms, including altered mental status and seizures, were more frequent in infections attributed to Omicron [81]. In addition, large-scale studies have indeed corroborated the fact that the incidence of PACS was highest among those infected with the original virus type but has gradually declined in its succeeding variants, including Omicron. But even so, fatigue seemed to remain the most common symptom in all variants, followed by pain, and then cognitive impairment [82].

Finally, cohort studies among pediatric populations have already shown differences among variants with respect to symptomatology. In fact, during the Omicron period, the respiratory and gastrointestinal manifestations significantly decreased in comparison to the period of previous variants, while increased severe neurological manifestations were recorded during this period. More specifically, there was an increase in altered mental status and seizures that were significantly higher in the Omicron period than in the non-variant and Alpha and Delta periods [83].

It is important to note that some contradictions exist across studies regarding the prevalence, severity, and types of PACS symptoms associated with different variants. These might have a biological underpinning, including but not limited to differences in virus–host interaction and immune response, and the psychosocial matters regarding the experience of the illness and perhaps the stress of living through the pandemic itself. Such complexities underscore the need for further research to reconcile these differences and provide a more comprehensive understanding of the factors shaping post-COVID-19 conditions.

### 4.7. Impact of Vaccination on PACS

Although the relationship between vaccination and PACS remains to be more deeply investigated in order to understand the underlying mechanisms, a portion of the literature has explored its protective effects. Specifically, statistics show that the probability of developing PACS is considerably lower among vaccinated people compared to unvaccinated people. One case, for example, identified vaccination to decrease the risk by about 29%, particularly after two vaccine doses. Another study found that only 8% of the vaccinated experienced post infection symptoms, compared with 27% of the unvaccinated [84,85].

Vaccination appears to protect against the severity of the symptoms, as well. Vaccinated persons report fewer severe respiratory symptoms, including shortness of breath, with overall alleviation of these symptoms with time. The protection may occur irrespective of whether vaccination has occurred before or after the SARS-CoV-2 infection, underlining the broad importance of vaccination in lowering the risk of post-COVID-19 conditions. Large population studies seem to support these findings. For example, a study in the UK showed that vaccinated people had a lower incidence of PACS symptoms, emphasizing the role of vaccination in lessening its impact. Interestingly, some patients with chronic symptoms also reported improvement after being vaccinated. All these disclosures underline the immense importance of vaccination, not only in the prevention of severe and acute cases of COVID-19, but also possibly in the alleviation of its long-term consequences [85,86].

## 5. The Psychological Dimensions of PACS

**Figure 3:**
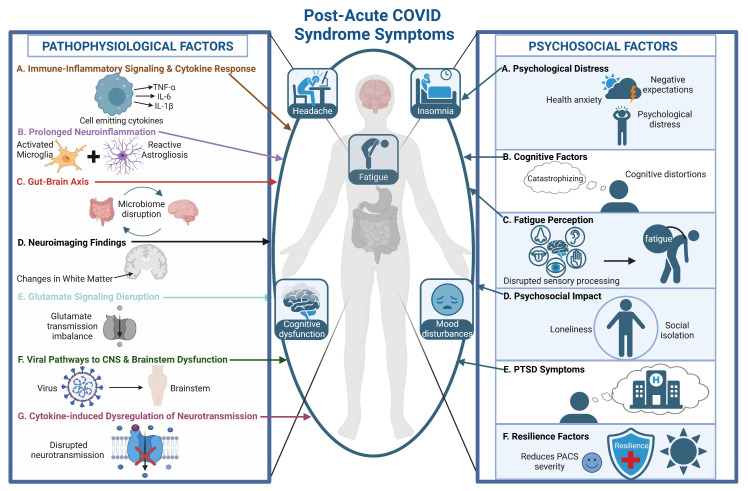
Integrative model of pathophysiological and psychosocial factors contributing to neurological symptoms in PACS (TNF-α: tumor necrosis factor-alpha; IL-6: interleukin-6; IL-1β: interleukin-1 beta; CNS: central nervous system; PACS: post-acute COVID-19 syndrome; PTSD: post-traumatic stress disorder).

So far, based on a very careful review of the literature available on this topic, we have been able to cover most of the symptomatology and psychopathology of the neurological profile of patients with PACS. In this chapter, we will examine these symptoms from both a psychological and psychosocial lens, taking into consideration not only the way in which these factors influence the course and experience of PACS but also the way in which they may be shaped by the condition itself (see Figure 3).

First of all, although a considerable portion of the current literature suggests that psychological factors contribute not only to the development but also to the ongoing maintenance of PACS, the influence of these psychological mechanisms is some-times overlooked or even dismissed—possibly due to concerns that acknowledging psychological factors could be perceived as diminishing the severity of the disease [87,88,89,90]. Within the academic community, the term “psychological” is often misinterpreted as something distinct from biological processes. However, in this review, we agree with the view almost collectively accepted within modern cognitive neuroscience, that psychological mechanisms are inherently brain-based and fundamentally integrated within the biological domain. In that sense, acknowledging the role of psychological factors does not imply a dismissal of the physiological complexities of PACS, but rather an expanded understanding of its multifaceted nature.

The research on persistent somatic symptoms and chronic medical conditions has highlighted the substantial role of psychological factors in these conditions. Given that PACS is often characterized by persistent somatic symptoms, these psychological mechanisms are likely relevant to this condition as well. Interestingly, health anxiety, negative expectations, and psychological distress frequently play a more critical role in reducing quality of life than the severity of somatic symptoms themselves. Other than depression and anxiety, cognitive perceptual factors such as selected attention, somatosensory amplification, and cognitive distortions—like catastrophizing—may further perpetuate symptom continuity [91,92,93].

As in many other chronic and somatic diseases, in PACS as well, it is presumed that psychological distress is not only a symptom but also a risk factor [44,94,95]. Other studies have found that psychological distress measured at the start of the pandemic was associated with increased risk of developing lasting symptoms among individuals who later became infected with SARS-CoV-2. Another interesting finding in the literature shows that in some cases, the expectation of COVID-19 symptoms and self-reported history of infection have been found to be stronger predictors of worsening somatic symptom burden compared to a laboratory-confirmed COVID-19 infection [96,97,98].

The intersection of somatic and psychological factors has recently driven Hüsing et al. to develop the PSY-PSS framework, a model designed to systematically examine psychological influences on the etiology of persistent somatic symptoms (PSSs). This framework provides a structured set of psychological variables that appear to aggravate symptomatology in individuals with PSSs. Key mechanisms include behavioral avoidance, where patients avoid situations that physically challenge the body, leading to inactivity and subsequent physical deconditioning. Cognitive factors, such as persistent rumination on physical complaints and a self-concept of bodily vulnerability, further contribute to symptom perpetuation. Additionally, affective traits—negative affectivity, anger linked to somatic experiences, and deficits in emotional regulation, particularly alexithymia—are central components in the PSY-PSS model. Without underestimating other physiological mechanisms, we can see the relevance of this psychological framework in understanding persistent somatic symptoms related to COVID-19 [92,93].

As we have seen thus far, fatigue is a common and disabling symptom in PACS, having a significant impact on daily functioning. Among other common factors, a model that could provide a valuable framework to further understand this persistent fatigue is what has been called the sensory attenuation model [99]. Under normal conditions, the brain suppresses self-generated sensory inputs—such as sensations from our own movements—to minimize unnecessary “noise” in perception, conserving energy and making physical tasks more efficient. This model does, however, suggest that once this mechanism is disrupted even simple movements could be more effortful and give rise to a sustained sense of fatigue. Indeed, the studies are suggesting that individuals with PACS could be experiencing disruption in the sensory process, thus giving them an increased sense of the feeling of effort and exhaustion in everyday tasks [100]. Unlike physical exhaustion alone, this is a type of fatigue that is produced by the changed way the brain handles the afferent input, which raises the perceived effort.

Arguably, fatigue is a symptom influenced by a variety of factors, and it can simultaneously contribute to the development of new symptoms. The positive feedback loop commonly observed in chronic diseases, where psychological and somatic manifestations interact and exacerbate each other, seems highly relevant to PACS as well. In this case, a vicious cycle is somewhat evident, as a somatic symptom triggers a psychological response, which in turn worsens the physical symptom or creates a new one, and so on. Interestingly, the cycle could also begin with a psychological trigger. Whatever the point of initiation of the circle is, however, it becomes clear that psychological symptoms could be powerful modifiers of the course of PACS. For instance, regarding the perception of fatigue in PACS, one study showed that patients who self-reportedly felt fatigued did not show additional objective fatigability compared to a control group without post-COVID-19 symptoms [101]. This could suggest that at least in some cases, the perception of fatigue in PACS may be more influenced by psychological factors than by objective measures of physical fatigue.

An interesting model that seems to align with the feedback loop mechanism described above was suggested by Scharfenberg et al. [102]. In this framework, the authors proposed that the SARS-CoV-2 infection may first induce symptoms like tiredness. Then, this could act as a central node, triggering a cascade of connected symptoms, via feedback loops within the network of symptoms. Next, the tiredness might lead to disturbed sleep, which could enhance the feelings of exhaustion. This persistent exhaustion may impair intellectual functioning, manifesting as difficulties with concentration, slowed processing speed, and a general sense of brain fog. These neuropsychological impairments may then enhance emotional suffering, which in turn increases symptoms like depression or anxiety. These neuropsychiatric symptoms could then feed back into the system, potentially escalating sleep disturbance and perpetuating the fatigue—thus completing a self-reinforcing cycle. The authors point out that this is merely one example of how symptoms could be interacting together, since these networks are very individualized and may vary quite a bit from person to person.

As we have seen in detail so far, the neuropsychological and neuropsychiatric consequences of COVID-19, particularly in the context of PACS, appear driven by a complex interplay of factors. These include the direct effects of the virus on the brain, as well as other indirect physiological influences. In the indirect pathway, we have already seen the psychological mechanisms that shape the course of the disorder. Beyond that, numerous studies have underlined the considerable psychosocial impact of the pandemic. Loneliness and social isolation, as well as the imposed restrictions during the pandemic, have been strongly associated with increasing levels of anxiety, depression, and other negative emotions, which could also increase the prevalence and severity of psychiatric symptoms among PACS patients [103,104,105,106]. Anxiety linked to the pandemic does not involve only the people who became infected with SARS-CoV-2. Anxiety disorders in the general population have also been found to present with similar physical symptoms to those of PACS, which, independent of the infection, would suggest that psychological distress may be implicated in symptom reporting [107]. On the contrary, higher personal resilience levels were linked to lower levels of PACS severity, therefore underlining the potential role of psychosocial factors in modulating symptom experience and recovery trajectories [89,108]. Similarly, neuropsychological problems, for instance, have been observed as persisting in the majority of cases, although other individuals seem to show higher rates of recovery, again pointing out that a combination of psychosocial and neuropsychological factors is at play [109,110].

A multitude of studies have shown that the gut–brain axis is critical in influencing and possibly precipitating the development of psychiatric and psychological disorders [111,112]. Furthermore, in our study, the hypothesis was highlighted that the gut–brain axis might also play a significant role in the pathogenesis of PACS itself. At this point, we interrupted the cycle to examine how stress can impact the gut–brain axis through its intricate bidirectional relationship. Sustained or severe stress can disrupt the activity of the vagus nerve—a key component of the gut–brain connection that regulates the parasympathetic nervous system. Decreased vagal tone, a reflection of the activity of the vagus nerve, can impair the ability of the body to recover from stress, maintain calmness, and support gastrointestinal and cardiovascular health. This disruption has the potential to impair gut function, leading to dysbiosis, and further exacerbating the dysfunction of the gut–brain axis, thus creating a feedback loop of physiological and psychological consequences of stress [113,114].

Finally, we have already highlighted that symptoms of PTSD, such as intrusive thoughts and hypervigilance, are common in PACS subjects. The symptomatology seems, for the most part, to be linked to the trauma of serious illness, hospitalization, the loss of a family member due to COVID-19, or even the fear of contracting the disease. The feedback loops persist in this domain as well, as the residual effects of PTSD, coupled with increased anxiety, seem to lead to sleep disturbances. About 26% of the PACS cases presented with prolonged sleep symptoms, including nightmares, which mapped the intensity of psychological stress onto nightly rest. Oneiric activity is consistent with the studies that have described patterns of dreams during the pandemic, suggesting continuity between wakeful and sleep mental activity [115,116,117].

## 6. Future Directions

At this point, we want to emphasize that the purpose of this study is not to underestimate the role of specific neurophysiological aspects in the course of PACS, as these are undeniably critical. Instead, the aim is to highlight the role of psychological and psychosocial factors—both in how they influence and are influenced by the disorder. For example, linking stress to worse outcomes in certain autoimmune conditions, such as rheumatoid arthritis, does not imply that the disease itself is psychogenic. In the same vein, acknowledging the influence of psychological elements such as anxiety or depression on PACS outcomes does not lessen the syndrome’s neurobiological reality but could add a new dimension to it that the healthcare community could utilize therapeutically.

### 6.1. The Role of Neuropsychological Assessments and Standardized Protocols

Throughout this review, it has become clear that the direct effects of the virus on the brain are, sometimes, very difficult to distinguish from the psychological and emotional repercussions of PACS. Neuropsychological assessments could play an essential role in navigating this complexity. While these evaluations cannot independently identify the underlying neuropathology of PACS, when combined with clinical expertise, they can contribute to a more comprehensive understanding of the condition. This combination could then allow clinicians to manage the neurological symptoms more effectively by also addressing the psychological and emotional toll of the disease.

Along these lines, a standardized approach to assessing the psychological aspects of PACS is also crucial. Utilizing a core set of neuropsychological and psychological instruments could improve the understanding of the relationship between psychological, biomedical, and social factors in the disease. Such tests could reveal the modifiable psychological processes, such as stress, cognitive distortions, and mood disorders, which are important targets for treatment strategies. Addressing these facets can enable healthcare providers to enhance the effectiveness of treatment and quality of life for the patients.

### 6.2. Psychological Interventions

The recent literature has determined the efficacy of psychological interventions, especially Cognitive Behavioral Therapy (CBT), to help patients with PACS manage some of their symptoms. One CBT protocol, modified for inpatient use, has shown promise in addressing psychological distress and persistent somatic symptoms associated with PACS. Those models commonly include structured group-therapy sessions focusing on cognitive restructuring, stress management, and illness-related coping strategies that are engaging psychophysiological mechanisms underlying somatic distress, with secondary attention to anxiety and depression, common comorbidities of PACS [118,119].

Expanding beyond traditional CBT approaches, interventions incorporating complementary techniques have shown promise in managing PACS-related psychological challenges. For example, combining CBT with mindfulness-based therapies, relaxation training, or eye movement desensitization and reprocessing has yielded additional advantages, especially in reducing emotional as well as somatic complaints. Dialectical behavioral therapy, focused on enhancing the regulation of emotions, has also proved to be useful for patients with PACS [120].

Another intervention that has shown positive results in the management of PACS is acceptance and commitment therapy, which focuses on enhancing psychological flexibility and acceptance. The studies have indicated that this kind of treatment may thus contribute to improving resilience and health-related quality of life in PACS patients, with the effects remaining up to several months after the treatment [121]. These findings, among others, show us the necessity of adaptive coping strategies when dealing with the complex psychological difficulties arising in patients with PACS.

Interpersonal and relational therapies, like interpersonal therapy and emotion-focused approaches, further provide frameworks for addressing the relational and emotional aspects of psychological suffering. These include relaxational interventions such as mindfulness and breathing to reduce anxiety and improve emotional resilience. Psychoeducation along with social support, meditation practices, therapies ranging from music therapy to narrative exposure therapy, and positive psychology interventions represent newer forms of therapy that have also been utilized in intervention strategies [122,123,124].

While these interventions have improved several symptoms in patients, more randomized controlled trials are much needed to establish the effectiveness of these treatments and for the optimization of their application in all aspects of diverse PACS.

### 6.3. Multidisciplinary and Patient-Centered Approaches

As we already mentioned, a portion of the current literature emphasizes the critical need for accessible, patient-centered, multidisciplinary healthcare provision. From healthcare professionals to family members and friends, ensuring that people with PACS are listened to and their experiences validated is paramount. Addressing the psychosocial aspects, which, as we saw, play a significant role in the prevalence of symptomatology, those responsible for healthcare policy and delivery should prioritize supporting individuals with PACS in adapting their work, social, and physical activities to align with their health capabilities. Helping them maintain or redefine their social functions and identities could be enormously beneficial as part of tailored occupational health programs. Finally, the impact of PACS on participation in social activities emphasizes the potentially huge value that tailored community groups and social prescribing hold, provided the programs are underpinned by ongoing research and evaluation to confirm their appropriateness and effectiveness.

### 6.4. The Role of AI in PACS Management

In light of the complexities involved in treating PACS, we already explored how a multidisciplinary approach is essential. Psychological support should be integrated into a comprehensive care model that also includes neurological treatments and cognitive rehabilitation. Integration of therapies such as CBT, together with cognitive rehabilitation and acceptance and commitment therapy, might provide comprehensive care that will address the psychological and cognitive impairments attributed to PACS. Symptom-matched and severity-matched interventions within a symptom management therapeutic model may enhance this care further in those patients whose symptoms remained in the mild category but were persistent enough to impact quality of life. Since we are living in the age of AI, its potential could provide valuable opportunities to enhance the management of PACS and its complexities. By integrating diverse data sources—such as electronic health records (EHRs), imaging, wearable sensors, and patient-reported outcomes—AI might support the development of more precise monitoring and intervention systems. Additionally, AI could contribute to more personalized care by leveraging data-driven insights to guide interventions tailored to each patient’s unique needs.

## 7. Discussion

The main goal of this review study was to explore the role of psychological, psychosocial, and neurobiological factors in the symptomatology of PACS. We have attempted to review the most recent neuropathological findings that lie behind the neurological and neuropsychiatric symptoms associated with PACS, in the context of how these might be modulated by psychological and psychosocial factors, which could in turn affect the severity and persistence of symptoms. By pointing out such complex interactions, we aim to contribute to the debate about how the development of an integrated treatment focused on neurobiological and psychological dimensions of PACS can be furthered.

Our findings suggest that psychological factors, such as stress, anxiety, and depression, along with psychosocial challenges arising from the aftereffects of quarantine measures, the isolation and loneliness experienced during the pandemic, and the pervasive fear and uncertainty surrounding health and recovery, are common among individuals with PACS. These factors appear to interact with the somatic manifestations of PACS in a way that creates a self-reinforcing cycle, where physical symptoms exacerbate psychological distress, which in turn worsens the physical symptoms. While this cycle is challenging to address, even small-scale interventions regarding psychological distress—such as CBT or supportive therapies—could help interrupt this loop. The partial interruption of this cycle could provide considerable relief and improvement in mental and physical health outcomes among affected individuals.

These findings seem to be compatible with a portion of the existing literature supporting the role of psychological distress in the exacerbation of symptoms of chronic diseases, especially those with a neurological component, including autoimmune diseases. However, the present study points out the need for a broader perspective. While much emphasis has been placed on the neurobiological aspects of PACS, we believe that psychological and psychosocial elements should be incorporated into treatment interventions in an attempt to possibly enhance holistic care. While further research is required to ascertain the effectiveness of these combined approaches, the evidence points to a promising direction for the development of PACS treatment.

In this context, we indicate the requirement for a multidisciplinary approach to PACS, in which psychological treatment strategies are considered along with neurobiological ones. Further research should focus on psychological interventions that reduce stress and anxiety in order to understand their potential in mitigating the exacerbation of symptoms. Longitudinal studies will further help in specifying the long-term effects of the interventions on neurological as well as psychological symptoms. Furthermore, it would be of value to identify how health care systems can support the development of models of care that address both the psychological and physical dimensions of PACS. Finally, newer technologies, such as artificial intelligence, could help in the identification of patterns and also support tailored intervention strategies.

Although this study may provide valuable insights, there are several limitations that must be stated. As in all research endeavors, there is always the possibility that some aspects of the literature may have been overlooked or that methodological flaws could create certain biased outcomes. Nevertheless, we believe this study represents a significant advance in understanding the complexities of PACS and indicating future research avenues. First of all, as previously mentioned, we spotted variability and sometimes conflicting results across different studies regarding the underlying causes of PACS and the effectiveness of various interventions. These inconsistencies may reflect differences in study design, population characteristics, or the evolving understanding of the condition. Second, the short duration since PACS has been identified as a distinct condition contributes to the difficulties in identifying absolute causal pathways or associations; much of the existing research is based on cross-sectional or early longitudinal data that may not fully capture the extended course of PACS or provide a comprehensive view of its long-term outcomes. Furthermore, the feedback loop created by psychosocial and pathophysiological elements, as has been elucidated in this paper, makes it sometimes difficult to have clear and conclusive endpoints. With that in mind, in this paper, it is acknowledged that our understanding of PACS is still evolving. However, the aim of our work is not to draw final conclusions but to add to the accumulation of knowledge about PACS and to discuss actionable strategies for harm reduction and improvements in patient outcomes. Further research must be defined into the diagnostic criteria, causal mechanisms, and optimal management strategies as more evidence comes in.

## 8. Conclusions

PACS is a complex disease with important health implications for patients and the medical infrastructure. Our review describes the complex combination of neurological, neuropsychological, and psychosocial influences on the longevity and complexity of PACS symptoms. It is clear that psychological stress, anxiety, and depression—as well as psychosocial difficulties like loneliness and uncertainty—can meld with neurobiological symptoms in a feedback loop to add an extra burden to both the physical and psychological suffering. While the understanding of PACS is still evolving, we want to highlight the importance of integrated, multidisciplinary approaches that address both biological and psychological dimensions. Among the promising areas for intervention are psychological interventions and holistic treatments, that attempt to break the cycle of symptom persistence. Although challenges remain, including variability and contradictions in research findings and the limitations of short-term data, in this work, the aim was to improve our knowledge of PACS and its management. Future research should prioritize longitudinal studies, refined diagnostic criteria, and innovative therapeutic strategies, contributing to a more comprehensive approach to PACS care and improving patient outcomes.

## Figures and Tables

**Table 1:** List of keywords used in database searches.

Keywords
Post-Acute COVID-19 Syndrome, PACS, PASC, Long COVID, Post-COVID, Pathology, Neuroinflammation, Brain Imaging, Brain Fog, Cognitive Dysfunction, Memory Impairment, Executive Function, Neuropsychiatric Symptoms, Neuropsychological Deficits, Quarantine Impact, Psychological Stress, Social Isolation, Loneliness, Mental Health, Fatigue, Headache, Insomnia, Depression, Immune Response, Autonomic Dysfunction, Viral Persistence

**Table 2:** Prevalence of neurological symptoms in PACS.

Neurological Symptom	Percentage of Affected Patients
Fatigue	Up to 58%
Sleep disturbances	Up to 30%
Headaches	Up to 44%
Dizziness and Vertigo	Up to 5%
POTS	Up to 14%
ME/CFS	Up to 51%
Gastrointestinal disturbances	Up to 22%

**Table 3:** Prevalence of neurocognitive symptoms in PACS.

Neurocognitive Symptom	Percentage of Affected Patients
Cognitive Impairment	Up to 65%
Persistent Fatigue	Up to 46.6%
Brain Fog	Up to 31.9%
Attention Deficits	Up to 55%
Memory Impairment	Up to 40%
Executive Function Deficits	Up to 59%
Language Difficulties	Up to 93%

**Table 4:** Prevalence of neuropsychiatric symptoms in PACS.

Neuropsychiatric Symptom	Percentage of Affected Patients
Anxiety	Up to 29.6%
Depression	Up to 35%
PTSD	Up to 16%
Psychotic Symptoms	Up to 4%
